# Micro1278 Leads to Tumor Growth Arrest, Enhanced Sensitivity to Oxaliplatin and Vitamin D and Inhibits Metastasis *via* KIF5B, CYP24A1, and BTG2, Respectively

**DOI:** 10.3389/fonc.2021.637878

**Published:** 2021-03-11

**Authors:** Weidong Lin, Heng Zou, Jinggang Mo, Chong Jin, Hao Jiang, Chengyang Yu, Zufu Jiang, Yusha Yang, Bin He, Kunpeng Wang

**Affiliations:** ^1^Department of General Surgery, Taizhou Central Hospital (Taizhou University Hospital), Taizhou, China; ^2^Taizhou Key Laboratory of General Surgery, Taizhou, China; ^3^Department of General Surgery, Second Xiangya Hospital, Central South University, Changsha, China

**Keywords:** micro1278, colorectal cancer, oxaliplatin, vitamin D, KIF5B, CYP24A1, BTG2

## Abstract

Colorectal cancer (CRC) is the most common cancer type in the digestive tract. Chemotherapy drugs, such as oxaliplatin, are frequently administered to CRC patients diagnosed with advanced or metastatic disease. A better understanding of the molecular mechanism underlying CRC tumorigenesis and the identification of optimal biomarkers for assessing chemotherapy sensitivity are essential for the treatment of CRC. Various microRNAs, constituting class of non-coding RNAs with 20-22 nucleotides, have served as oncogenes or tumor suppressors in CRC. We analyzed miR-1278 expression in clinical samples by qRT-PCR. We then explored the role of miR-1278 in CRC growth *in vitro* and *in vivo* as well as sensitivity to oxaliplatin *via* RNA-seq and gain- and loss-of-function assays. We found that miR-1278 was downregulated in CRC samples, correlating with advanced clinical stage, and overexpression of miR-1278 led to tumor growth arrest and increased sensitivity to oxaliplatin *via* enhanced apoptosis and DNA damage. Suppression of KIF5B by miR-1278 through direct binding to its 3′UTR was the mechanism for the miR-1278-mediated effects in CRC, miR-1278 inhibits metastasis of CRC through upregulation of BTG2. Additionally, we also found that the expression of CYP24A1, the main enzyme determining the biological half-life of calcitriol, was significantly inhibited by miR-1278, according to data from clinical, RNA-seq and functional assays, which allowed miR-1278 to sensitize CRC cells to vitamin D. In summary, our data demonstrated that miR-1278 may serve as a potential tumor suppressor gene and biomarker for determining sensitivity to oxaliplatin and vitamin D in CRC.

## Introduction

Although the incidence of colorectal cancer (CRC) has decreased every year since 2003, it remains the most diagnosed cancer in the digestive system worldwide (1, 2). In China, the new cases of CRC increased at a rate of ~1% between 2006 and 2010 (3). The molecular mechanism underlying CRC progression has greatly advanced; nevertheless, surgery is the only modality for radically removing cancer (4). Chemotherapy involving oxaliplatin is preferred for advanced or metastatic disease. Although an overall survival increases through chemotherapy, relatively limited patients benefit from it, and predictive markers suggesting individual benefits of chemotherapy are still scarce for patients with CRC (5). Thus, a better understanding of the molecular mechanisms underlying tumorigenesis and the screening of appropriate biomarkers for chemotherapy response are essential for CRC patients.

MicroRNAs are a class of non-coding RNAs with 20-22 nucleotides that play an essential role in cell proliferation, differentiation, death and other bioprocesses *via* specific binding with the 3′UTR (untranslated regions) of targeted genes, resulting in mRNA translational inhibition or degradation (6). Since the initial validation of microRNAs, such as let-7 (which is vital for the developmental biology of *Caenorhabditis elegans*), microRNAs have been demonstrated to be dysregulated in various diseases, but the underlying functions and mechanisms are unknown (7, 8). Many studies have reported that microRNAs are actively involved in tumorigenesis, progression and drug response in various types of cancers, such as brain, lung, breast, pancreatic cancer and CRC (9–13). The expression of microRNAs is frequently altered in cancers due to mutations, deletion amplification, transcriptional changes or post-transcriptional modifications (14, 15). Thus, many studies have extensively investigated the clinical significance, biological functions and therapeutic indications of these microRNAs in cancers. However, the microRNA signature of cancer is largely influenced by the types of tissues, progression status and therapeutic conditions (16).

In CRC, various microRNAs serve as oncogenes or tumor suppressors and participate in tumor growth and invasion, maintaining stemness, apoptosis, autophagy and therapeutic response or resistance (17, 18). On account of the stability within clinical samples and the expressive abundance, microRNAs exhibit greater utility than mRNAs for use as predictors of prognosis, classification of disease and auxiliary therapy decisions. For instance, miR-375 is highly expressed in CRC tissues compared to peritumoral tissues, and higher expression of miR-375 correlates with lymph node metastasis and distant metastasis (19). miR-34a is essential for interleukin-6-induced epithelial-to-mesenchymal transition in CRC by directly targeting the STAT3 transcription factor, which promotes phenotypic transition, invasion and metastasis (20). For the regulation of therapeutic response and resistance, overexpression of miR-143 improves oxidative stress and apoptosis of oxaliplatin-resistant CRC cells after oxaliplatin treatment, while miR-195 desensitizes CRC cells to some genotoxic drugs (21, 22). In addition, the advancement of genomics and new high-throughput sequence profiling technologies has led to a large increase in microRNA data pertaining to various statuses of CRC, in which the significant heterogeneity of microRNA expression will be largely unveiled. Thus, an understanding of the clinical significance and functional indication of the novel microRNAs is needed.

Recent works suggested an essential role of a novel microRNA, miR-1278 in tumor progression of glioma and drug sensitivity in nasopharyngeal carcinoma, while the function of it in CRC remains elusive (23, 24). In our work, we found that the expression of miR-1278 was significantly downregulated in CRC tissues compared to peritumoral tissues and negatively correlated with T stage and TNM stage. Overexpression of miR-1278 led to tumor growth arrest *in vitro* and *in vivo* as well as sensitization to oxaliplatin by increasing DNA damage and apoptosis by binding with the 3′UTR of KIF5B, a member of the kinesin family in CRC. In addition, overexpression of miR-1278 increased the sensitivity of CRC cells to vitamin D, which showed a robust antitumor effect on CRC, by suppressing the expression of CYP24A1, the main enzyme determining the biological effect of vitamin D. Furthermore, miR-1278 mimic led to inhibition of metastasis by upregulation of BTG2. Thus, we provided pre-clinical evidence to demonstrate that miR-1278 may be beneficial for improving the therapeutic response to oxaliplatin and vitamin D in CRC patients.

## Materials and Methods

### Agents

Oxaliplatin and SDZ285-428 were purchased from TargetMol (USA).

### Human Samples and CRC Cell Lines

This study was authorized by the Ethics Committee of Taizhou Central Hospital (Taizhou University Hospital) and was performed according to the approved guidelines. Written consent was obtained when using the patient's surgical specimen. The cancerous tissues and paracancerous tissues of 42 patients diagnosed with colorectal cancer were collected after surgical resection and preserved at −80°C. The HT29, HCT116, and SW620 CRC cell lines were genotyped by STR and obtained from ZSBIO (China). Cells were cultured in RPMI-1640 medium supplemented with 10% fetal bovine serum and 1% penicillin-streptomycin. Cells were cultured in a humidified incubator at 37°C and 5% CO_2_.

### Cell Viability/Proliferation Assay

Cells in logarithmic growth phase were seeded into 96-well plates, cultured in a 5% CO_2_ incubator at 37°C for 24 h and then cultured for 3 h after adding CCK-8 reagent (GeneView, USA) to each well. The OD value was measured at 450 nm by a microplate spectrophotometer (Thermo Fisher, USA). The experiment was repeated three times. All values were normalized to the control group (without adding cells), and the results are presented as the mean ± SD.

### Colony Formation Assay

After transfection, diluted CRC cells were seeded in a 6-well plate at a concentration of 5,000 cells per well. After 7 days of culture, the cells were washed twice with PBS, fixed with 4% paraformaldehyde and stained with 1% crystal violet (Beyotime, China), and colony images were captured with a digital camera.

### Subcutaneous Xenograft Model

SPF-grade BALB/c nude mice were purchased from the Experimental Animal Center of Central South University. All animal experiments followed the ethical principles of animal experiments and were performed in strict accordance with operating standards. HCT116 cells transfected with miR-1278 mimic or control were subcutaneously injected into the backs of 6-week-old male mice with a total number of 2 × 10^6^ cells. After 4 weeks of standard feeding, the mice were sacrificed, and tumor samples were collected.

### Western Blot Analysis

Colorectal cancer specimens and colorectal cancer cells were lysed with RIPA buffer (TargetMol, USA) containing protease inhibitor and phosphatase inhibitor. The supernatant was collected after centrifugation at 4°C for 30 min. After the protein was quantified and denatured, the denatured protein was separated by SDS-PAGE electrophoresis and then transferred to a PVDF membrane. After blocking the membrane with 3% bovine serum albumin for 1 h, primary antibody was added and incubated overnight at 4°C. After removal of the primary antibody, the membrane was washed three times with TBST and incubated with secondary antibody at room temperature for 1 h. Immune complexes were detected by an enhanced chemiluminescence system (Life Tec, USA), and protein bands were analyzed and quantified using ImageJ software (version 11). The primary antibodies were as follows: caspase 3 (Abclonal, China), caspase 9 (Abclonal, China), cleaved caspase 3 (CST, USA), CYP24A1 (CST, America), cleaved caspase 9 (Abclonal, China), γh2A (Abclonal, China), Ki67 (Abclonal, China) and GAPDH (Abclonal, China). The secondary antibody was purchased from Abclonal (China).

### Quantitative Real-Time PCR

RIPA lysate was added to colorectal cancer tissue and colorectal cancer cells, and samples were lysed at 4°C for 10 min. After centrifugation at 12,000 g, RNA was extracted with isopropanol, and the purity and concentration of RNA were detected by a Nanodrop 2000 spectrophotometer (Thermo Fisher, USA). A cDNA reverse transcription kit (Life Tec, USA) was used to synthesize cDNA according to the instructions. The following primers were used: KIF5B, 5′- CAACCGCAATTGGAGTTATAGG-3′ (forward) and 5′- ATTCAGCTCAGCTTGCATATTG−3′ (reverse); and CYP24A1, 5′-AAAGTATCTGCCTCGTGTTGTA-3′ (forward), 5′-CTTCTCTAACCGGTTGTCGATA-3′ (reverse). For qRT-PCR, 2× Universal SYBR Green Fast qPCR mix (Abclonal, China) and a LightCycler 96 system (Roche, USA) were used.

### Immunohistochemical Staining

The paraffin wax was cut into 4-μm-thick sections, heated at 66°C for 30 min, dewaxed and hydrated in xylene solution and gradient ethanol solution. Sections were then incubated for 15 min in 3% H_2_O_2_ formaldehyde solution to seal and repair antigen, and the corresponding primary antibody was added and incubated at 37°C for 60 min. Sections were then washed three times with PBS, and an appropriate amount of secondary antibody was added and incubated at 37°C for 30 min. An appropriate amount of DAB solution was added, and sections were stained and restained with hematoxylin, and neutral gum was then added. Cover slides were sealed with coverslips and observed under a microscope.

### Immunofluorescence

Colorectal cancer cells were cultured in 24-well plates for 24 h, washed three times with PBS and fixed with 4% paraformaldehyde. Cells were blocked with 3% bovine serum albumin, and the corresponding primary antibody was then added and incubated overnight at 4°C. After washing three times with PBS, the secondary antibody was added and incubated at room temperature for 60 min in the dark. Cells were washed and stained with DAPI (Invitrogen, USA), and immunofluorescence signal was detected by a fluorescence microscope (Olympus Inc., USA).

### EdU Assay

Colorectal cancer cells cultured in 24-well plates for 24 h were washed with phosphate. EDU was mixed with cell culture medium and added to cells followed by incubation at 37°C for 120 min. After 3 min of washing with phosphate, cells were permeabilized with 0.25% Triton X-100, stained with DAPI and imaged by fluorescence microscopy (Olympus, USA).

### Plasmid Transfection

Cells were seeded in a 6-well plate and cultured for 24 h at 50% confluence. The corresponding plasmid and negative control group were transfected into cells with Lipofectamine™ 2000 Transfection Reagent (Invitrogen, USA). The KIF5B sequence was cloned into the GV141 vector. After incubation for 48 h, cell function was analyzed.

### siRNA Transfection

For transfection, the cells were seeded in 6-well plates at 50% confluence. After 24 h of incubation, cells were transfected with siRNAs or negative controls using Lipofectamine™ RNAiMAX transfection reagent (Invitrogen). The targeted sequence of BTG2 siRNA was AGGGAGCAAGCAAGGTTAGC and the siRNA was synthesized by Ribobio, China. After another incubation for 36 h, the cells were subjected to the assay as indicated. The knockdown efficiency of the siRNA was confirmed by qRT-PCR and Western blot analysis.

### Cell Migration

Cell migration was determined by Transwell assay (Corning, America). In brief, 2 × 10^5^ cells were seeded in the inserts resuspended by 250 ul RPMI 1640 medium and placed into the 24-well plates containing 500 ul RPMI 1640 medium supplemented with 10% FBS and incubated for 24 h at 37°C with 5% CO_2_. The cells in the insert that did not migrate through the pore were carefully removed by scraping with wet cotton swab. The migrated ones were fixed in 4% paraformaldehyde for 10 min and stained with 0.1% crystal violet solution (Beotime, China).

### Flow Cytometry Assay

The apoptosis rate of colorectal cancer cells was detected by an Annexin V-FITC/PI apoptosis detection kit. Each group of cells was resuspended in 100 μl of Annexin V-FITC binding buffer containing 5 μl of Annexin V-FITC and 5 μl of PI staining solution followed by incubation in the dark at room temperature for 10 min. Then, 400 μl of binding buffer was added to each sample and mixed. Apoptosis was analyzed by flow cytometry (Becton Dickinson, USA).

### Luciferase Assay

The plasmid involved in the luciferase experiment was synthesized by Shanghai Jikai Gene Co., Ltd. According to the instructions of the Invitrogen Lipofectamine 2000 transfection reagent, the Dual-Glo^®^ Luciferase Buffer and Dual-Glo^®^ Stop & Glo^®^ Buffer were incubated at room temperature 3 days in advance. Then, 250 μl of medium was removed from each well of a 24-well plate, and 250 μl of Dual-Glo^®^ Luciferase reagent was added to the well and reacted at room temperature for 10 min. Then, the reagent was aspirated, and the cells were centrifuged. It was confirmed that the cells were fully lysed, and they were transferred to a 1.5-mL EP tube. Then, 100 μl was absorbed into the Lockwell MaxiSorp detection plate, and firefly luminescence was detected by an enzyme labeling instrument. Then, 50 μl of Dual-Glo^®^ Stop & Glo^®^ Reagent was added to each well for 30 min at room temperature, and fluorescence was detected.

### Alkaline Comet Assay

Colorectal cancer cells were cultured for 24 h, treated with oxaliplatin, digested with trypsin for 1 min and centrifuged, and the supernatant was discarded. Cells were lysed, washed three times with PBS, soaked in alkaline unwinding solution, dehydrated and stained with DAPI solution after electrophoresis using an agarose gel. The experimental results were observed under a fluorescence microscope.

### RNA-Sequencing

A total RNA amount of 1 μg per sample was used as input material for the RNA sample preparations. Sequencing libraries were generated using the NEBNext^®^ UltraTM RNA Library Prep Kit. After cluster generation, the library preparations were sequenced using an Illumina NovaSeq platform, and 150 bp paired-end reads were generated. The RNA-sequencing procedure and data analysis were performed by Novogene, China.

### Statistical Analysis

All statistical analyses were performed using Statistical Product and Service Solutions (SPSS) software version 22.0 and Prism software (GraphPad Prism 6). Student's *t*-test was applied to assess significant differences between two groups. For three or more groups, one-way ANOVA was used. All statistical analyses were performed using a two-sided test. *P*-values < 0.05 were considered statistically significant.

## Results

### miR-1278 Is Downregulated in CRC, and the miR-1278 Mimic Inhibits Tumor Growth *in vitro* and *in vivo*

**Figure 1:**
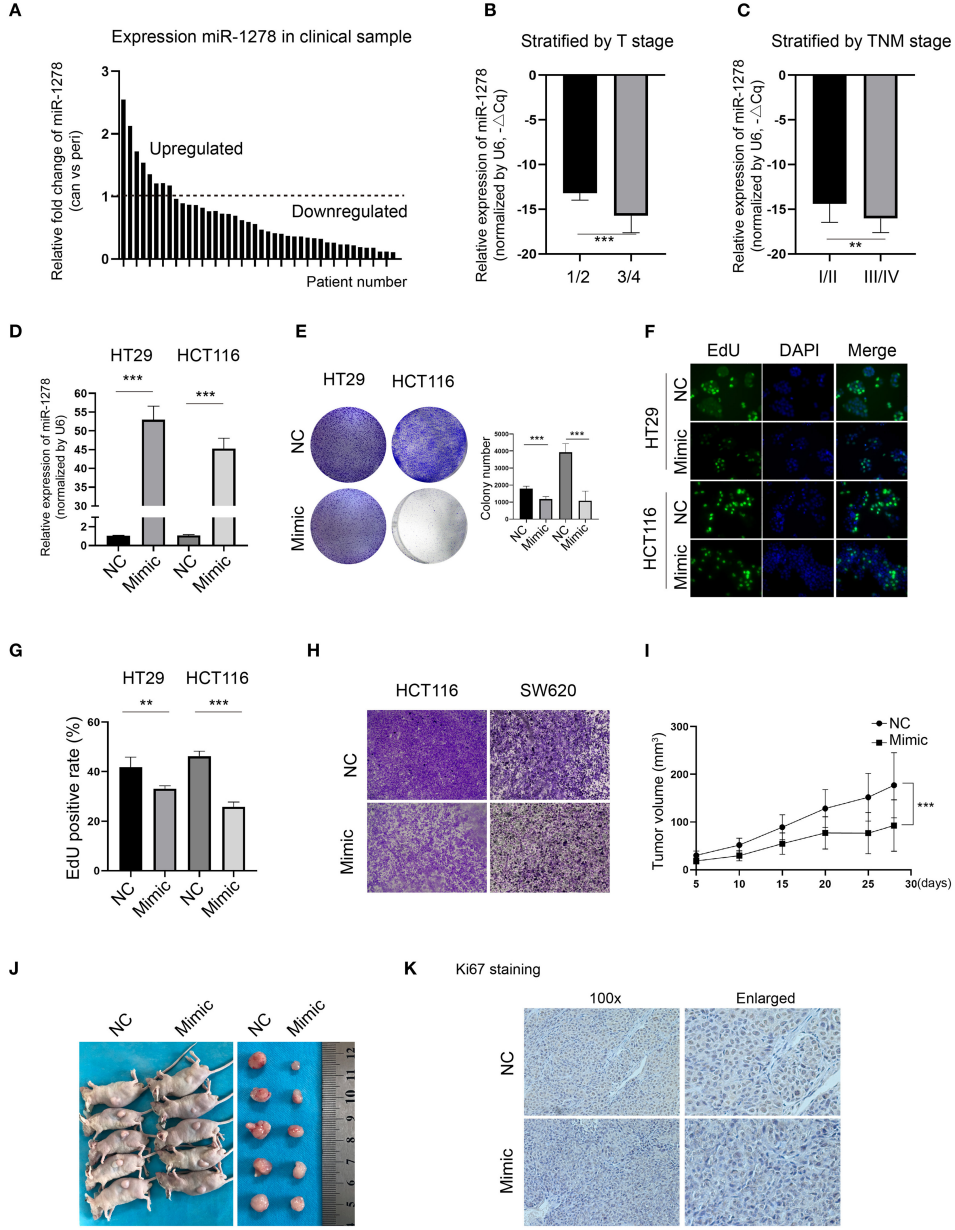
miR-1278 is downregulated in CRC, and the miR-1278 mimic inhibits tumor growth *in vitro* and *in vivo*. **(A)** The expression of miR-1278 was determined in 42 pairs of CRC samples and the associated peritumoral tissues by qRT-PCR. miR-1278 was downregulated in 34 pairs, while it was upregulated in 8 pairs. **(B,C)** Relative expression of miR-1278 in CRC tissues compared to the reference gene. ΔCq refers to the Cq of the target gene minus that of the reference gene; thus, –ΔCq represents the miR-1278 expression level normalized to GAPDH. When stratified by T stage, high grade was associated with low expression of miR-1278. When stratified by TNM stage, high grade was correlated with low expression of miR-1278. **(D)** HT29 and HCT116 cells were transfected with the miR-1278 mimic and verified by qRT-PCR. **(E)** A colony formation assay was performed to determine the colony formation ability. Transfection of the miR-1278 mimic reduced colony formation in HT29 and HCT116 cells. **(F,G)** The EdU assay showed that transfection of the miR-1278 mimic significantly inhibited cell proliferation in HT29 and HCT116 cells. **(H)** Transwell assays showed that transfection of the miR-1278 mimic greatly impaired the migration of HCT116 and SW620 cells. **(I)** HCT116 cells transfected with miR-1278 or control were subcutaneously injected into nude mice followed by one injection at a 3-day interval. Tumor sizes were measured every 5 days. **(J)** After ~4 weeks, mice were sacrificed, and tumors were collected. **(K)** IHC staining of Ki67 demonstrated that Ki67 was downregulated in the miR-1278 mimic group compared to the control group. NC, negative control; Mimic, miR-1278 mimic; **P < 0.01, ***P < 0.001.

To explore the expression of miR-1278 in CRC, we collected and determined its expression in a cohort of 42 pairs of CRC samples and the corresponding peritumor samples. Compared to the peritumor samples, miR-1278 was downregulated in most tumor samples (*n* = 34), while it was upregulated in 8 tumor samples (Figure 1A). When stratified by T stage, miR-1278 was significantly decreased in higher grades (3/4) than in lower grades (1/2) (Figure 1B). A similar result was also observed after stratification by TNM stage (Figure 1C). To delineate the potential role of miR-1278 in CRC, miR-1278 was overexpressed in two CRC cell lines, HT29 and HCT116, *via* transfection of the miR-1278 mimic (Figure 1D). Colony formation assays showed that the miR-1278 mimic markedly inhibited colony formation in HT29 and HCT116 cells (Figure 1E). The EdU assay further verified that the miR-1278 mimic significantly decreased the cell proliferation rate in both cell lines (Figures 1F,G). In addition, the miR-1278 mimic also led to impaired cell migration in HCT116 and SW620 cells, which have robust invasion ability (Figure 1H). We used a subcutaneous tumor model to evaluate the *in vivo* role of miR-1278 and found that the miR-1278 mimic significantly decreased tumor volume compared to the negative control (Figures 1I,J). Consistently, the miR-1278 mimic reduced the positive rate of Ki67 staining in tumor samples compared to the control (Figure 1K). Thus, these data demonstrated that miR-1278 is downregulated in CRC and that aberrant expression of miR-1278 leads to tumor growth arrest *in vitro* and *in vivo*.

### miR-1278 Mimic Sensitizes CRC Cells to Oxaliplatin by Inducing Apoptosis

**Figure 2:**
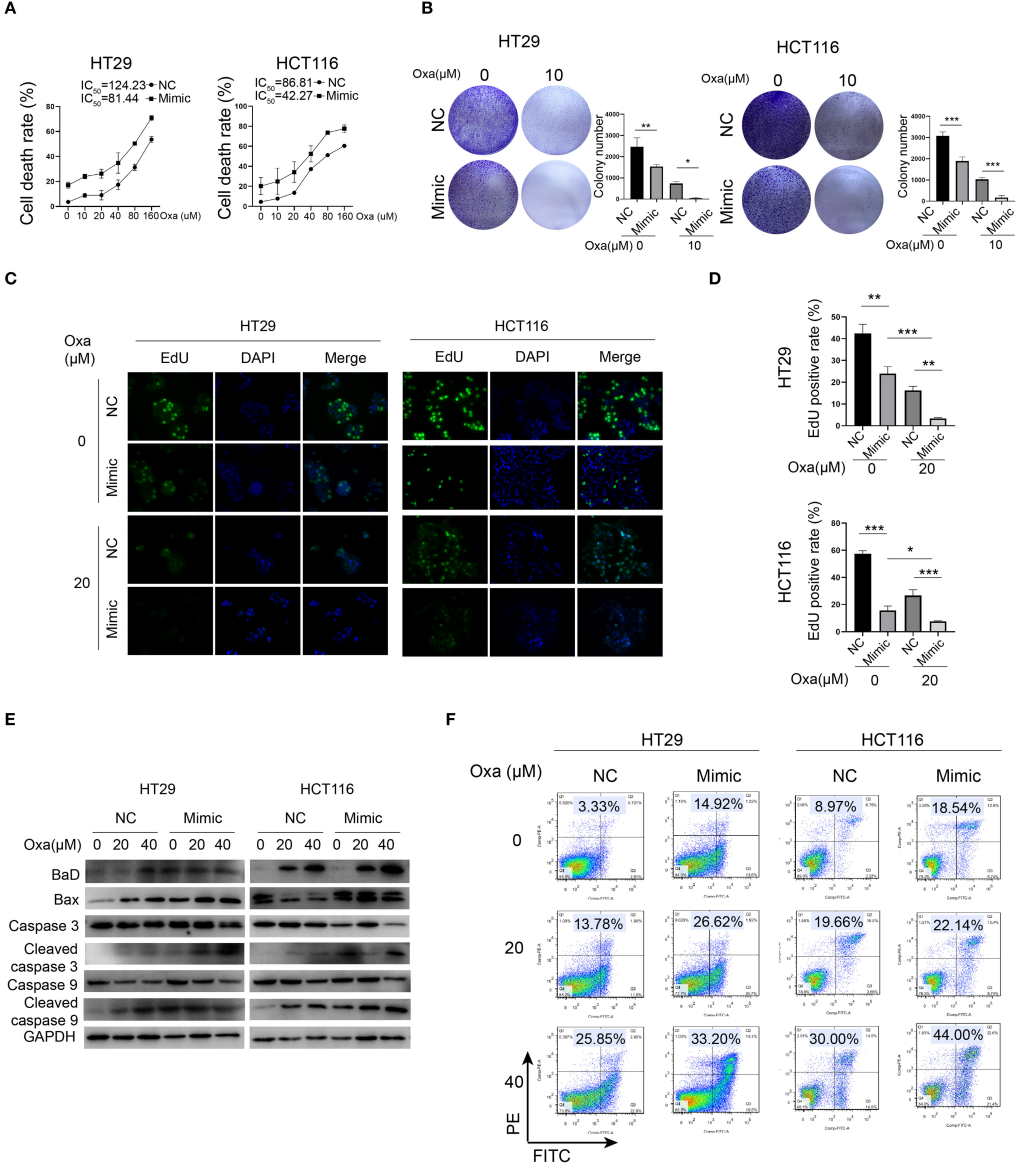
miR-1278 mimic sensitizes CRC cells to oxaliplatin by inducing apoptosis. **(A)** Cell viability was detected by CCK-8 assay in HT29 and HCT116 cells treated with various concentrations of oxaliplatin for 24 h. The IC50 values of the NC and miR-1278 mimic groups were 124.23 and 81.44 μM, respectively, in HT29 cells as well as86.81 and 42.27 μM, respectively, in HCT116 cells. **(B)** Cells (5,000 cells per well) were seeded and cultured for 3 days, and cells were then treated with oxaliplatin and cultured for another 4 days. The results showed that the miR-1278 mimic greatly inhibited colony formation of HT29 and HCT116 cells treated with 10 μM oxaliplatin. **(C,D)** Transfection of miR-1278 significantly reduced the proliferation of HT29 and HCT116 cells treated with 20 μM oxaliplatin as demonstrated by the EdU assay. **(E)** Comparison of the expression levels of caspase 3, cleaved caspase 3, caspase 9, cleaved caspase 9 and Bax and Bad between the miR-1278 mimic and control groups treated with various concentrations of oxaliplatin is shown as indicated. These proapoptotic proteins were concomitantly upregulated in the miR-1278 mimic group compared to the control group, indicating that transfection of the miR-1278 mimic facilitates apoptosis induced by oxaliplatin in CRC cells. **(F)** Flow cytometry was performed to quantitatively detect the proportion of apoptosis in each group. The apoptosis rates were 26.62 and 32.20% when HT29 cells were treated with 20 and 40 μM oxaliplatin, respectively, in the miR-1278 mimic group as well as 13.78 and 25.85% in the control group when HT29 cells were treated with 20 and 40 μM oxaliplatin, respectively. The same trend was also observed in HCT116 cells. Oxa, oxaliplatin; NC, negative control; mimic, miR-1278 mimic; *P < 0.05, **P < 0.01, ***P < 0.001.

Because oxaliplatin is a preferred chemotherapy drug for CRC patients, we investigated whether miR-1278 influences the sensitivity of CRC cells to oxaliplatin. Compared to the control group, the miR-1278 mimic markedly promoted oxaliplatin-induced cell death in HT29 cells (half maximal inhibitory concentration or IC_50_ was 124.23 vs. 81.44 μM) and HCT116 cells (IC_50_ was 86.81 vs. 42.27 μM) (Figure 2A). Consistently, oxaliplatin markedly inhibited colony formation in HT29 and HCT116 cells, and the miR-1278 mimic impaired colony formation in both cell lines (Figure 2B). The EdU assay showed that the miR-1278 mimic promoted the inhibitory effect of oxaliplatin on cell proliferation (Figures 2C,D). Moreover, we detected the expression of various apoptosis-associated proteins, including Bad, Bax, cleaved caspase 3 and cleaved caspase 9, in HT29 and HCT116 cells treated with oxaliplatin with or without miR-1278 mimic. As shown in Figure 2E, the miR-1278 mimic markedly improved the expression of Bad, Bax, cleaved caspase 3 and cleaved caspase 9 when treated with the same concentration of oxaliplatin compared to the control group, indicating enhanced apoptosis. Furthermore, when treated with 20 μM oxaliplatin, the apoptosis rates of the control and mimic groups were 13.78 and 26.62% in HT29 cells as well as 19.66 and 22.14% in HCT116 cells, respectively (Figure 2F). The apoptosis rates increased from 25.85 to 33.20% in HT29 cells and from 30.0 to 44.0% in HCT116 cells after treatment with 40 μM oxaliplatin. In summary, these data showed that aberrant expression of miR-1278 markedly improves the sensitivity of CRC cells to oxaliplatin by inducing apoptosis.

### KIF5B and CYP24A1 Expression Is Inhibited by the miR-1278 Mimic in CRC Cells

**Figure 3:**
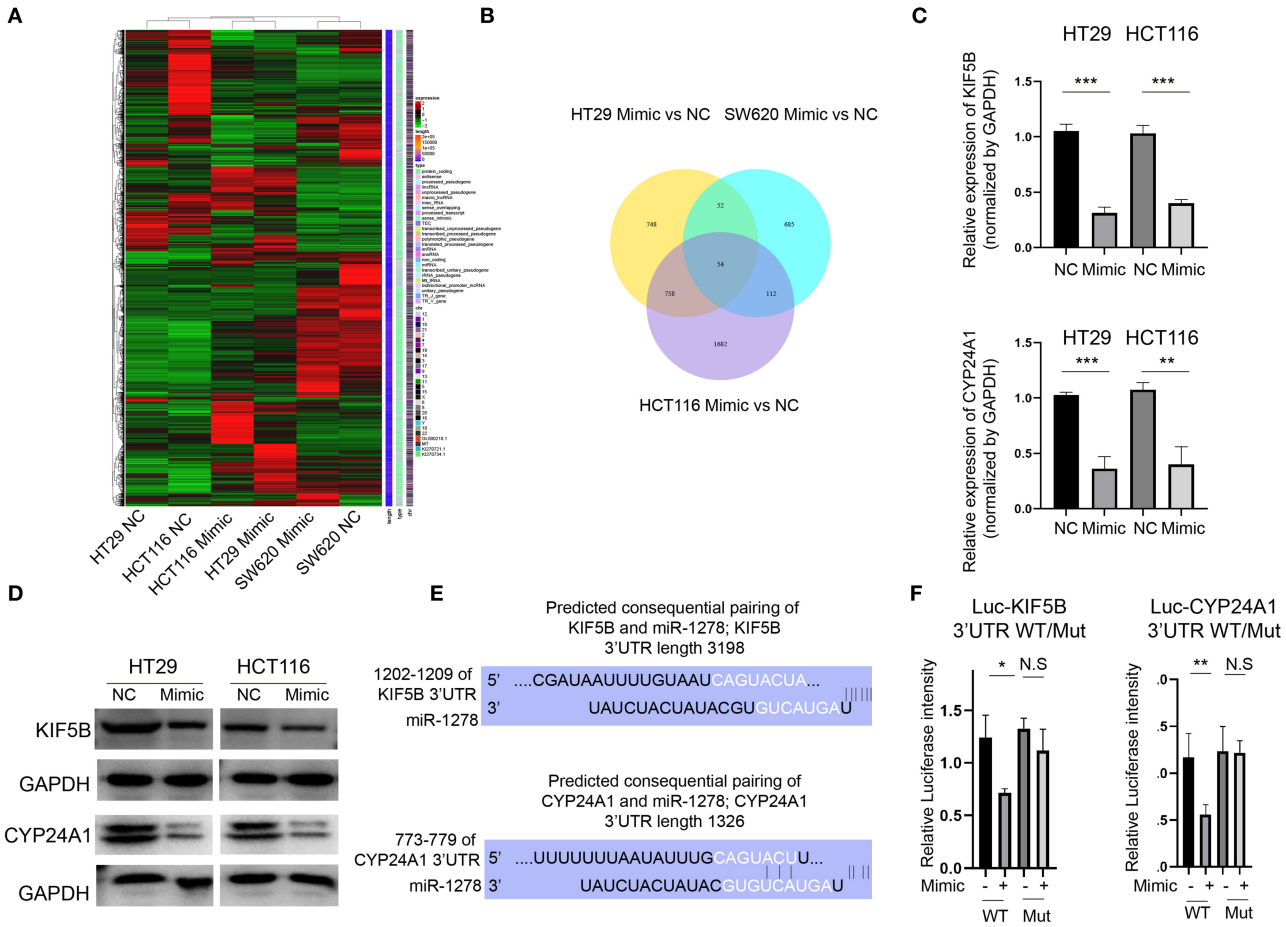
KIF5B and CYP24A1 expression is inhibited by the miR-1278 mimic in CRC cells. **(A)** The miR-1278 mimic was transfected into three CRC cell lines (HT29, HCT116 and SW620), and RNA-seq was performed to compare whole gene expression variations between the mimic group and the control group. **(B)** In total, 1,612 genes, 2,606 genes, and 903 genes were screened at an absolute fold change value > 2 in HT29, HCT116 and SW620 cells, respectively. Only 54 genes consistently differed among the three cell lines. **(C)** Among them, two genes, KIF5B and CYP24A1, were selected for further analysis. The mRNA expression of KIF5B and CYP24A1 was verified by qRT-PCR in HT29 and HCT116 cells. **(D)** The protein expression of KIF5B and CYP24A1 was determined by Western blotting, indicating that the miR-1278 mimic markedly decreased the protein expression of KIF5B and CYP24A1 in HT29 and HCT116 cells. **(E)** The predicted consequential pairing of miR-1278 and the 3′UTR of KIF5B or CYP24A1 was determined. **(F)** The luciferase reporter assay showed that miR-1278 directly binds with the KIF5B 3′UTR and CYP24A1 3′UTR. NC, negative control; Mimic, miR-1278 mimic; WT, wild type; Mut, mutated; *P < 0.05, **P < 0.01, ***P < 0.001, N.S, no significance.

To clarify the downstream regulators of miR-1278 in CRC cells, we analyzed the global gene expression variations by RNA-seq in three CRC cell lines (HT29, HCT116 and SW620) after transfection with the miR-1278 mimic (Figure 3A). A cutoff value of fold change = 2 showed that 1,612, 903, and 2,606 genes were dysregulated in HT29, SW620 and HCT116 cells, respectively, and 54 genes commonly differed among the three cell lines (Figure 3B). We focused on KIF5B, which is a key protein for the central spindle during cytokinesis, and CYP24A1, a vitamin D-metabolizing enzyme. We confirmed that the mRNA expression of KIF5B and CYP24A1 in HT29 and HCT116 cells was significantly decreased after transfection with the miR-1278 mimic (Figure 3C). The protein expression of KIF5B and CYP24A1 was also markedly inhibited by the miR-1278 mimic (Figure 3D). We then predicted the consequential pairing of miR-1278 and KIF5B or CYP24A1 by the TargetScan online tool (Figure 3E). Using a luciferase reporter assay, we further verified that miR-1278 directly binds to the KIF5B 3′UTR and CYP24A1 3′UTR, while not binds to the mutated ones (Figure 3F). Collectively, our data demonstrated that miR-1278 negatively regulates the expression of KIF5B and CYP24A1 by directly binding with the 3′UTR.

### KIF5B and CYP24A1 Are Overexpressed in CRC and Negatively Correlated With the Expression of miR-1278

**Figure 4:**
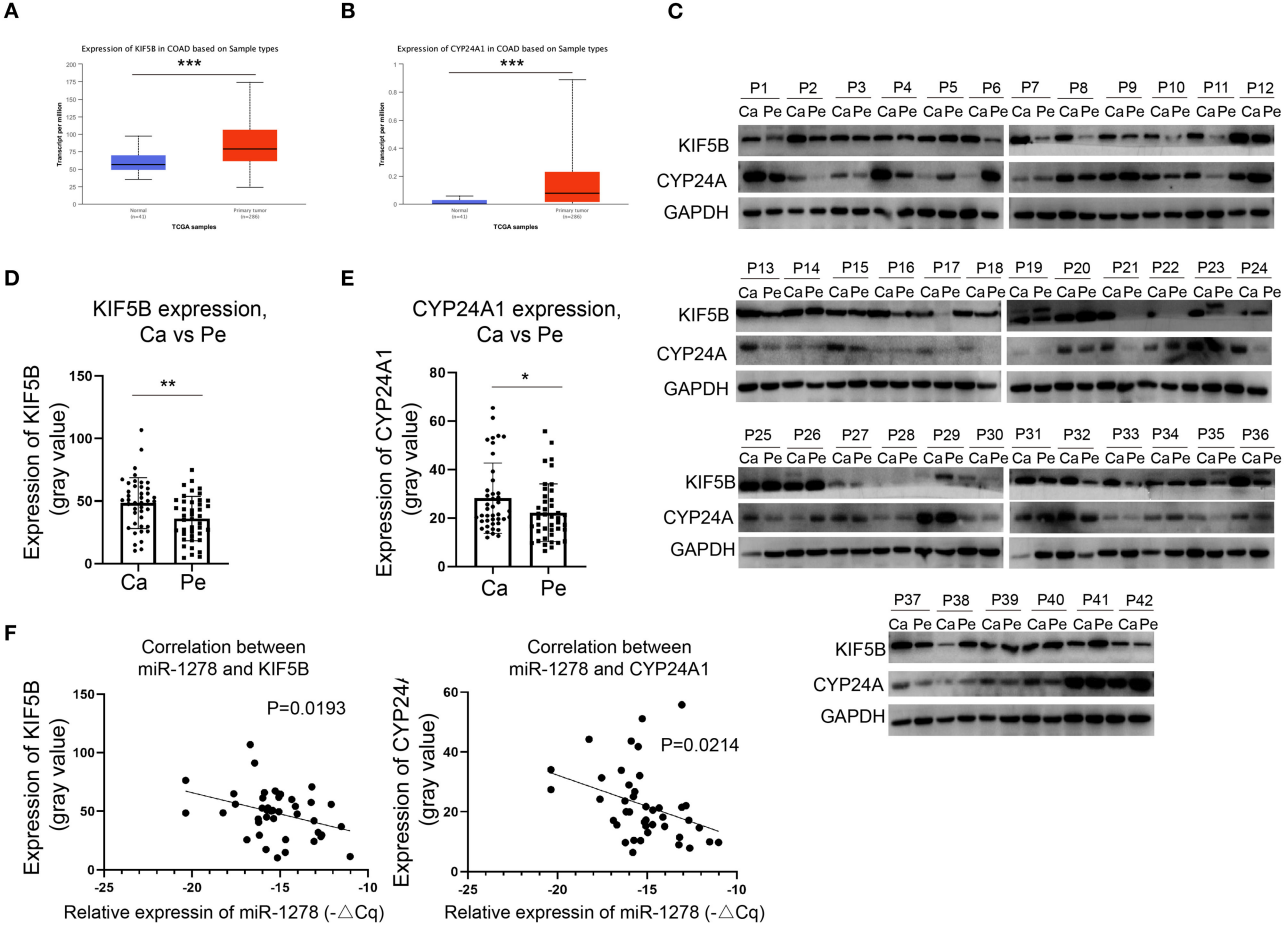
KIF5B and CYP24A1 are overexpressed in CRC and negatively correlate with the expression of miR-1278. **(A,B)** The expression of KIF5B and CYP24A1 was measured in datasets of the TCGA COAD (colon adenocarcinoma) program. KIF5B and CYP24A1 were significantly upregulated in tumor samples compared to normal tissues. **(C)** The protein expression of KIF5B and CYP24A1 was determined by Western blotting in 42 pairs of CRC samples. **(D)** Compared to peritumoral tissues, KIF5B was significantly upregulated in cancer tissues. **(E)** Compared to peritumoral tissues, CYP24A1 was significantly upregulated in cancer tissues. **(F)** KIF5B or CYP24A1 expression and miR-1278 showed a negative correlation in CRC cancer tissues. Ca, cancer; Pe, peritumoral; P, patient; *P < 0.05, **P < 0.01.

We demonstrated that miR-1278 was downregulated in CRC and that KIF5B and CYP24A1 might be essential downstream regulators. We next investigated whether the expression of KIF5B or CYP24A1 was upregulated in CRC and correlated with the expression of miR-1278. Based on the data from the TCGA COAD program, we found that KIF5B and CYP24A1 were significantly overexpressed in tumor samples compared to normal tissues (Figures 4A,B). We measured the protein expression of KIF5B and CYP24A1 in 42 pairs of CRC samples, and the results are presented in Figure 4C. These findings demonstrated that the expression of KIF5B and CYP24A1 was significantly upregulated in CRC tissues compared to peritumoral tissues (Figures 4D,E). Moreover, the protein expression of either KIF5B or CYP24A1 was negatively associated with the expression of miR-1278 in CRC tissues (Figure 4F). Thus, our data showed that the potential downstream targets of miR-1278, namely KIF5B and CYP24A1, are upregulated in CRC and negatively associated with the expression of miR-1278.

### Downregulation of KIF5B Is Critical for the miR-1278 Mimic-Mediated Sensitization to Oxaliplatin by Promoting DNA Damage and Apoptosis

**Figure 5:**
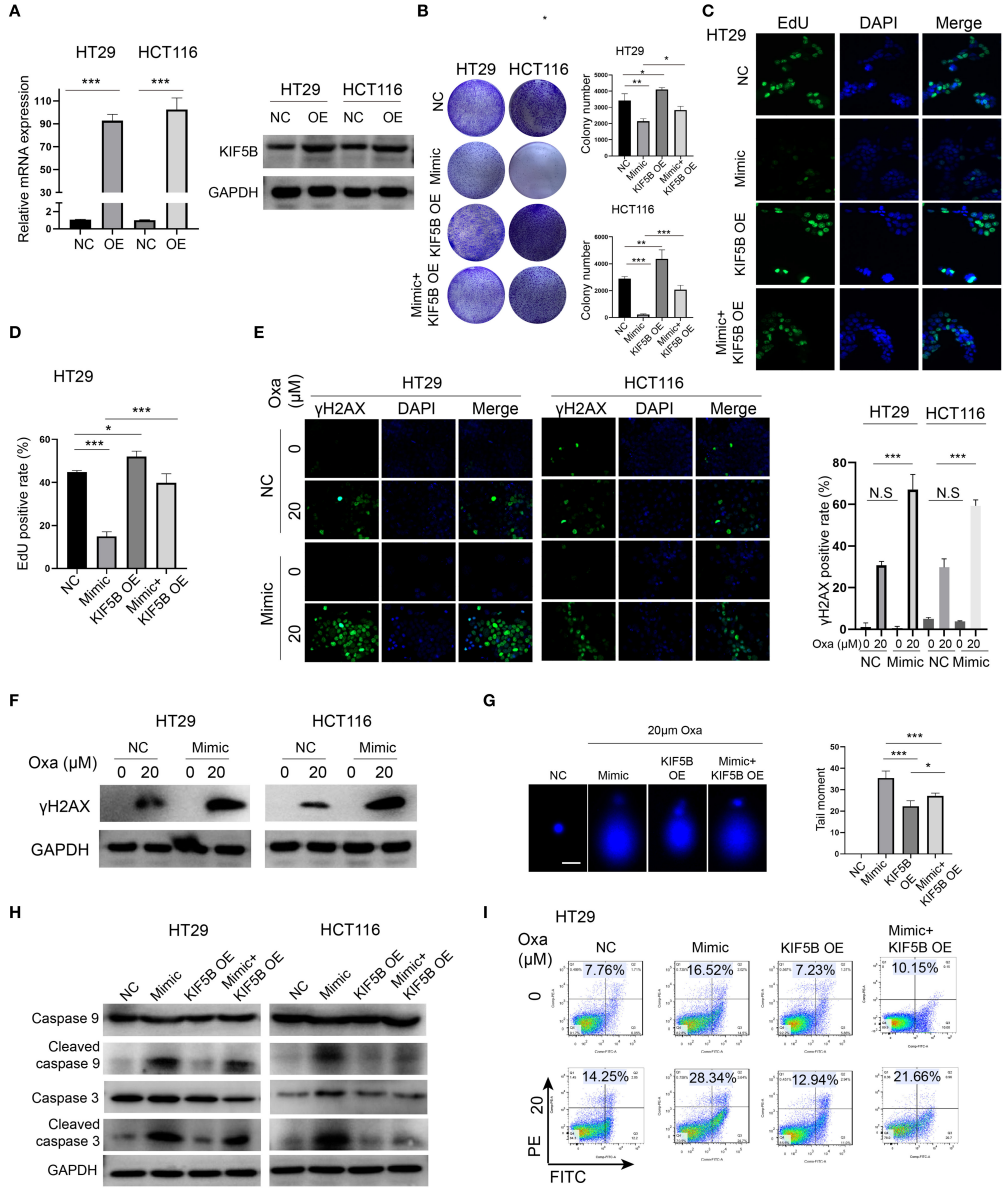
Downregulation of KIF5B is critical for the miR-1278 mimic-mediated sensitization to oxaliplatin by promoting DNA damage and apoptosis. **(A)** KIF5B was overexpressed using a plasmid in HT29 and HCT116 cells as verified by qRT-PCR and Western blot. **(B)** The colony formation assay demonstrated that overexpression of KIF5B rescued the inhibitory effect of the miR-1278 mimic on colony formation in HT29 and HCT116 cells. **(C,D)** Overexpression of KIF5B significantly rescued the inhibitory effect of the miR-1278 mimic on HT29 cell proliferation. **(E,F)** γh2AX served as a sensitivity marker for DNA damage and was detected by immunofluorescence and Western blotting. DNA damage was greatly enhanced in cells transfected with the miR-1278 mimic after oxaliplatin treatment. **(G)** An alkaline comet assay was used to detect single-stranded and double-stranded DNA damage. The results showed that overexpression of KIF5B markedly rescued miR-1278-mediated DNA damage after oxaliplatin treatment by decreasing single-stranded and double-stranded DNA damage. **(H)** Detection of the expression levels of caspase 3, cleaved caspase 3, caspase 9 and cleaved caspase 9 in the control, miR-1278 mimic, KIF5B overexpression and miR-1278+KIF5B overexpression groups. Overexpression of KIF5B rescued miR-1278-mediated apoptosis after oxaliplatin treatment in HT29 and HCT116 cells. **(I)** Overexpression of KIF5B markedly decreased the apoptosis rate from 28.34% in the miR-1278 group to 21.66% after 20 μM oxaliplatin treatment in HT29 cells. NC, negative control; Mimic, miR-1278 mimic; Oxa, oxaliplatin; *P < 0.05, **P < 0.01, ***P < 0.001; N.S, no significance.

We overexpressed KIF5B by transfection of the KIF5B WT plasmid, and we verified that its mRNA and protein expression was significantly upregulated in HT29 and HCT116 cells (Figure 5A). Overexpression of KIF5B abrogated the miR-1278 mimic-mediated inhibition of colony formation in HT29 and HCT116 cells (Figure 5B). The EdU assay verified that KIF5B overexpression led to increased cell proliferation and that it largely abrogated the effect of the miR-1278 mimic on cell proliferation (Figures 5C,D). Previous studies have suggested that several members of the kinesin family are intimately correlated with chemotherapy-induced DNA damage in cancers. We tested whether the miR-1278 mimic causes enhanced DNA damage in PC cells. γh2AX, which is the phosphorylated form of H2AX and correlates well with double-strand breaks (DSBs) of DNA, serves as the most sensitive marker for DNA damage. The miR-1278 mimic did not yield enhanced DNA damage in the absence of oxaliplatin. However, in the presence of oxaliplatin, overexpression of miR-1278 significantly accelerated DNA damage in HT29 and HCT116 cells (Figures 5E,F). Moreover, the alkaline comet assay also demonstrated that the miR-1278 mimic markedly induced DBS or single-strand breaks in the presence of oxaliplatin, while KIF5B overexpression prevented DNA damage induced by oxaliplatin (Figure 5G). Interestingly, overexpression of KIF5B largely abrogated the miR-1278 mimic-mediated apoptosis after treatment with oxaliplatin, which was represented by decreased expression of cleaved caspase 3 and cleaved caspase 9 in the mimic+KIF5B overexpression group compared to the mimic group (Figure 5H). KIF5B overexpression decreased the apoptosis rate from 28.34 to 21.66% in the mimic group in the presence of oxaliplatin (Figure 5I). Collectively, these data demonstrated that overexpression of KIF5B abrogates the miR-1278 mimic-mediated sensitization to oxaliplatin by preventing DNA damage and apoptosis.

### miR-1278 Sensitizes CRC Cells to Vitamin D by Suppressing the Expression of CYP24A1

**Figure 6:**
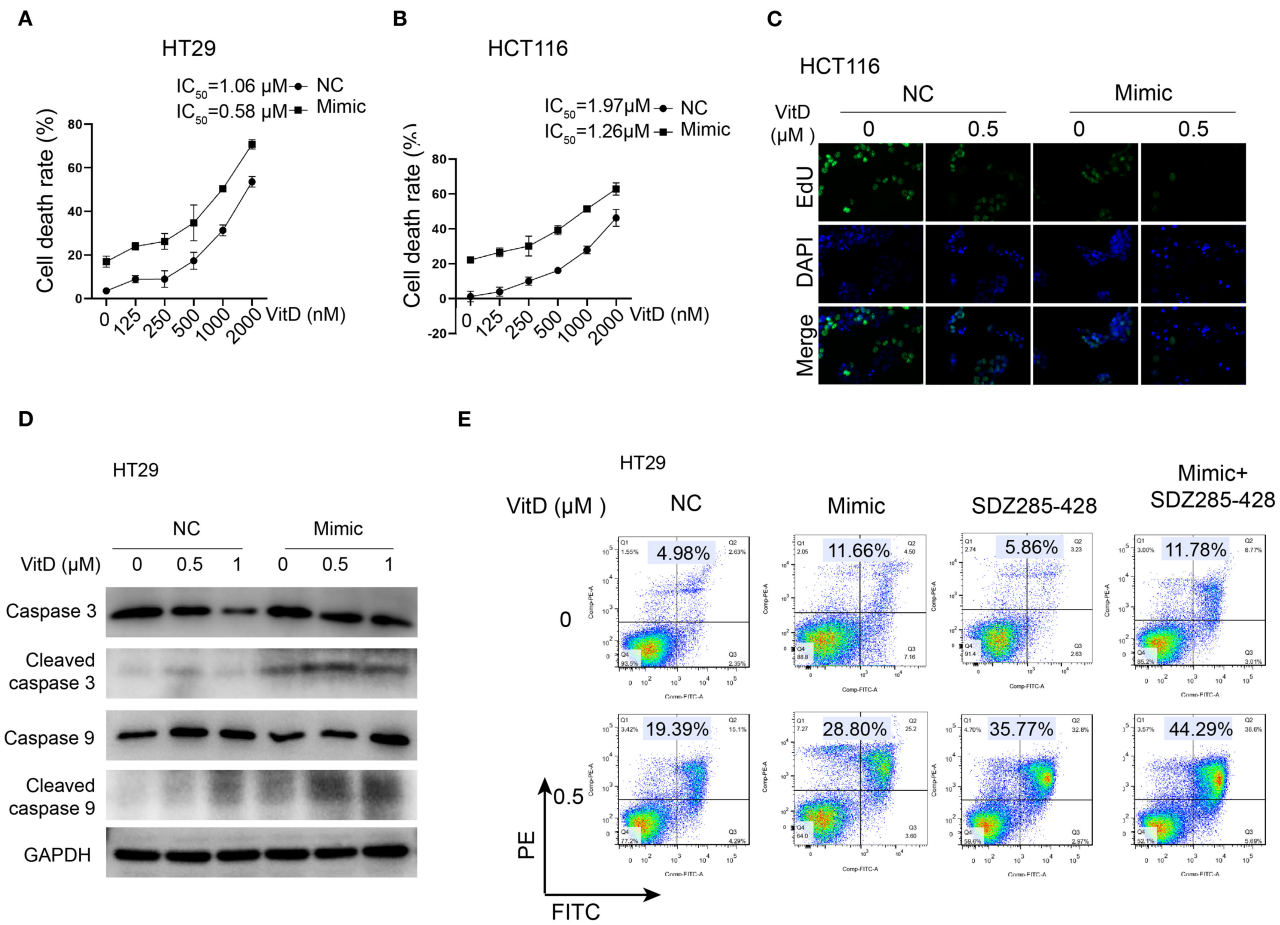
miR-1278 sensitizes CRC cells to vitamin D by depressing the expression of CYP24A1. **(A,B)** Cell viability was detected by CCK-8 assay in HT29 and HCT116 cells treated with various concentrations of vitamin D for 24 h. The IC50 values of the NC and miR-1278 mimic groups were 1.06 and 0.58 μM, respectively, in HT29 cells and 1.91 and 1.26 μM, respectively, in HCT116 cells. **(C)** Transfection of miR-1278 markedly inhibited the proliferation of HCT116 cells treated with 0.5 μM vitamin D as demonstrated by the EdU assay. **(D)** The expression of cleaved caspase 3 and cleaved caspase 9 was increased in the miR-1278 mimic group compared to the control group after treatment with the same concentration of vitamin D. **(E)** SDZ285-428 is a specific inhibitor of CYP24A1. Either the miR-1278 mimic or SDZ285-428 led to an improved apoptosis rate after vitamin D treatment, and the combination treatment of the miR-1278 mimic and SDZ285-428 resulted in a further enhancement of apoptosis in HT29 cells. VitD, Vitamin D; NC, negative control; Mimic, miR-1278 mimic.

Because CYP24A1 was demonstrated as a targeting protein of miR-1278, we investigated whether overexpression of miR-1278 facilitates CRC cell death after treatment with vitamin D. The IC_50_ of vitamin D decreased to 0.58 and 1.26 μM in the miR-1278 mimic group compared to 1.06 and 1.97 μM in the control group in HT29 and HCT116 cells, respectively (Figures 6A,B). The EdU assay showed that the miR-1278 mimic markedly inhibited cell proliferation after treatment with the same concentration of vitamin D (Figure 6C). Furthermore, the miR-1278 mimic induced higher expression of cleaved caspase 3 and cleaved caspase 9 after vitamin D treatment in HT29 cells compared to the control group (Figure 6D), indicating that miR-1278 facilitates vitamin D-induced apoptosis. SDZ285-428 is a specific inhibitor of CYP24A1. Flow cytometry assays showed that the miR-1278 mimic combined with SDZ285-428 did not increase the apoptosis rate in the absence of vitamin D compared to the mimic group but that it increased apoptosis after treatment with vitamin D (Figure 6E). These data suggested that miR-1278 sensitizes CRC cells to vitamin D, possibly *via* a CYP24A1-dependent pathway.

### miR-1278 Inhibits Migration Through Upregulation of BTG2

**Figure 7:**
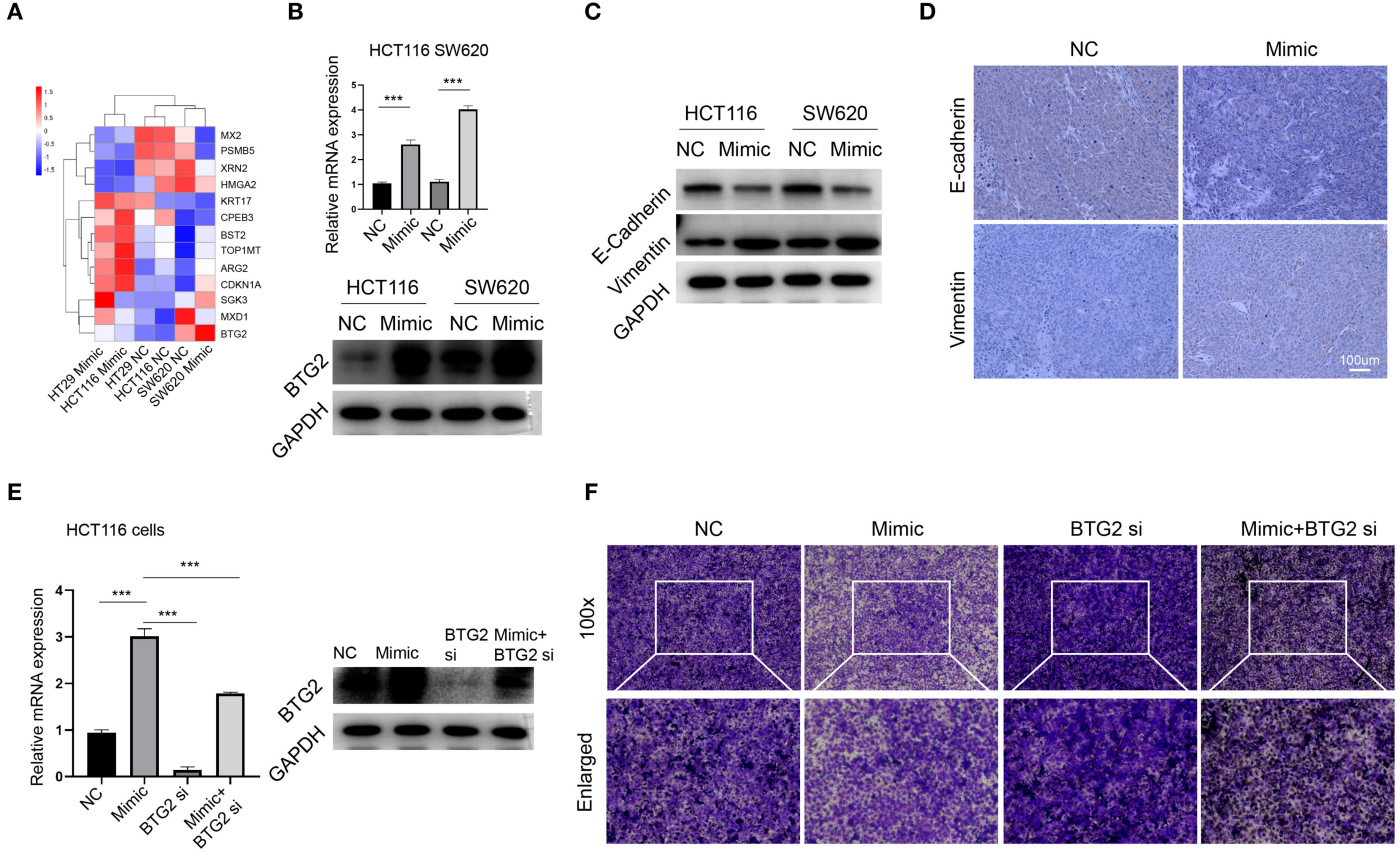
miR-1278 inhibits migration through upregulation of BTG2. **(A)** Compare the expression of 13 genes consistently altered in three CRC cell lines presented by heat map. **(B)** The mRNA expression of protein expression of BTG2 was determined by qRT-PCR and Western blotting in HCT116 and SW620 cells. **(C,D)** Western blot analysis and IHC staining of e-cadherin and vimentin demonstrated that EMT was inhibited in the miR-1278 mimic group compared to the control group. **(E)** qRT-PCR and Western blot showed that BTG2 siRNA successfully knockdown BTG2 and rescued the expression of it promoting by miR-1278 mimic in HCT116 cells. **(F)** BTG2 siRNA largely rescued the inhibition of migration exerted by miR-1278 mimic in HCT116 cells. NC, negative control; Mimic, miR-1278 mimic; si, BTG2 siRNA; ***P < 0.001.

Our initial data suggested that miR-1278 mimic inhibited migration of CRC cells, and we further explored the potential mechanism. Among the genes consistently altered in HT29, HCT116, and SW620 cells, 13 genes had previously been reported associated with tumor metastasis, such as BTG2, MXD1, CDKN1A, and HMGA2. We focused on BTG2, the most changed genes between mimic and control groups (Figure 7A). The results from RNA-seq was verified by qRT-PCR and Western blot assay (Figure 7B), collectively demonstrated that miR-1278 significantly improved the expression of BTG2, an essential gene for inhibition of metastasis. Epithelial-mesenchymal transition (EMT) was conferred by loss of BTG2 in various cancer (25, 26). Our data further showed that miR-1278 mimic inhibited EMT process *via* markedly promoting the expression of vimentin while downregulating e-cadherin in CRC cells and tumor samples from xenograft model (Figures 7C,D). Administration of BTG2 siRNA largely rescued the expression of BTG2 (Figure 7E) and improved the migration of HCT116 cells (Figure 7F) impaired by miR-1278 mimic. Thus, miR-1278 mimic-mediated inhibition of migration was performed by upregulation of BTG2 in CRC.

## Discussion

MicroRNAs are emerging as an abundant class of endogenous non-coding RNAs that are extensively understudied as disease biomarkers and active regulators of bioprocesses in cancers. Numerous dysregulated microRNAs have been unveiled in CRC, such as miR-155, miR-18a, miR-7, and miR-30a, which are downregulated in CRC compared to normal tissues, as well as miR-494, miR-221, and miR-214, which are upregulated in CRC compared to normal tissues (17). MicroRNAs have been identified as appropriate biomarkers to discriminate tumor metastasis status (27). Several microRNAs have also been demonstrated to be valuable for predicting tumor recurrence in stage II and stage III CRC patients (28). In the present study, we evaluated a novel microRNA, miR-1278, in CRC based on a cohort of samples and found that miR-1278 was significantly downregulated in CRC compared to peritumoral tissues and was negatively correlated with T stage and TNM stage. Forced expression of miR-1278 impaired tumor growth and inhibited tumor migration. These data emphasized the clinical significance of miR-1278 in CRC and suggested its potential role in tumorigenesis in CRC.

Oxaliplatin is an essential chemotherapy drug used for CRC patients with advanced or metastatic disease (29, 30). Following the aquation process, the reactive form of oxaliplatin binds DNA bases to generate DNA adducts and DNA damage (31). Mounting evidence has suggested that microRNAs are valuable determiners of oxaliplatin sensitivity and crucial for the regulation of the DNA damage response in cancer (32). miR-625-3p, miR-181b, and miR-27b have been demonstrated to be correlated with the response to combined therapy involving oxaliplatin in a cohort of 26 patients (33). Another study has shown that low expression of miR-4299 and high expression of miR-196b indicate a preferred overall survival in colon cancer patients who received combined therapy involving oxaliplatin (34). A recent study has verified that miR-1278 is significantly downregulated in nasopharyngeal carcinoma and that aberrant expression of miR-1278 leads to high sensitivity to cisplatin (24). In our analysis, miR-1278 was also suppressed in CRC samples compared to peritumoral tissues, and the miR-1278 mimic markedly improved sensitivity to oxaliplatin *via* elevated apoptosis and DNA damage. MicroRNAs actively participate in the regulation of the DNA damage response after exposure to genotoxic chemotherapies with a molecular mechanism involving DNA damage sensing, signal transducing and DNA damage repair (35). Based on multiple lines of evidence, we found that the miR-1278 mimic greatly improved DNA damage in CRC cells treated with oxaliplatin. Mechanistically, miR-1278 deregulated the expression of KIF5B, a member of kinesins ubiquitously expressed in tissues that is essential for transporting organelles, membranous vesicles and other cargoes (36), by specifically targeting its 3′UTR. The role of some kinesins in the regulation of DNA repair and DNA stability has been elucidated, and it is exemplified by KIF4, which mediates DNA replication and DNA repair processes by interacting with DNA and DNA-binding proteins to maintain chromatin and chromosome structure (37). However, it remains unknown how KIF5B participates in the DNA damage response after oxaliplatin treatment.

Apart from the well-recognized physiological role of vitamin D in regulating calcium and phosphate metabolism, recent evidence has suggested a robust antitumor effect of vitamin D, especially in CRC. Vitamin D is activated to calcitriol by two hydroxylation steps, and CYP24A1 is the main enzyme determining the biological half-life of calcitriol (38). Previous studies have shown that CYP24A1 mRNA expression is not only upregulated in CRC cell lines but also in CRC samples, especially in poorly differentiated and late-stage cancers (39). Our analysis further demonstrated that CYP24A1 protein expression was well-correlated with that of mRNA expression reported by others. CRC cell viability and proliferation are largely unaffected by calcitriol alone but are significantly reduced when the activity of CYP241A is simultaneously inhibited (40). These observations suggest that targeting CYP24A1 might be the key pathway to improve the sensitivity of CRC to vitamin D. MicroRNAs have been implicated in the antitumor effect of vitamin D. For instance, calcitriol upregulates miR-627, which further suppresses the expression of KDM3A, a histone demethylase resulting in inhibition of cell proliferation by negatively regulating several proliferation-associated genes (41). miR-22 is induced by calcitriol and regulates various target genes of calcitriol, which is critical for its antitumor effect in CRC cells (42). However, research concerning the role of microRNAs in regulating the expression of vitamin D metabolism-associated genes has not been reported. Our study demonstrated that miR-1278 suppresses CYP24A1 expression in CRC cells and sensitizes CRC cells to treatment with vitamin D.

Importantly, a previous report has shown that coadministration of vitamin D analogs and cisplatin leads to an enhanced inhibitory effect in cancer cells *via* modulation of the cell cycle and ROS production (43). The synergistic effect of vitamin D with chemotherapy has been further verified by an *in vivo* model (44). In our analysis, we provided evidence to elucidate the role of miR-1278 in potentiating the sensitivity of CRC cells to oxaliplatin and vitamin D. However, it remains unknown whether miR-1278 is beneficial for combination therapy of oxaliplatin and vitamin D in CRC.

In summary, our study demonstrated a tumor suppressor role of miR-1278, which suppresses tumor growth and migration as well as improves sensitivity to oxaliplatin and vitamin D in CRC. We further identified the member of the kinesin family, KIF5B, as a targeted downstream target of miR-1278 through specific binding with its 3′UTR, resulting in augmented DNA damage and apoptosis, and miR-1278 inhibits metastasis of CRC through upregulation of BTG2. In addition, we also found that miR-1278 suppresses the expression of CYP24A1, a main enzyme determining the biological half-life of calcitriol, which is critical for the elevated sensitivity to vitamin D in CRC. However, a better understanding of the potential role of miR-1278 in potentiating the efficacy of the combination therapy of oxaliplatin and vitamin D is required in future work.

## Data Availability Statement

The raw data supporting the conclusions of this article will be made available by the authors, without undue reservation.

## Ethics Statement

The studies involving human participants were reviewed and approved by Ethics Committee of Taizhou Central Hospital (Taizhou University Hospital). The patients/participants provided their written informed consent to participate in this study. The animal study was reviewed and approved by Ethics Committee of Taizhou Central Hospital (Taizhou University Hospital).

## Author Contributions

KW and WL conceived of the project, performed most of the experiments, and writing the manuscript. KW, HZ, and JM participated in the experimental design and data analysis. CJ, HJ, CY, ZJ, YY, and BH collected clinical samples, provided financial support, and valuable suggestions for the revision of manuscripts. All authors contributed to the article and approved the submitted version.

## Conflict of Interest

The authors declare that the research was conducted in the absence of any commercial or financial relationships that could be construed as a potential conflict of interest.
